# Low birth weight: prevalence and associated factors among newborns at hospitals in Kambata-Tembaro zone, southern Ethiopia 2018

**DOI:** 10.11604/pamj.2019.34.68.18234

**Published:** 2019-10-02

**Authors:** Abebe Alemu, Mulatu Abageda, Biruk Assefa, Getnet Melaku

**Affiliations:** 1 Department of Midwifery, College of Health Science and Medicine, Wachamo University Hosana, Ethiopia; 2 Department of Midwifery, College of Health Science and Medicine, Dilla University, Dilla, Ethiopia

**Keywords:** Low birth weight, newborn, southern Ethiopia

## Abstract

**Introduction:**

More than 20 million infants were born with low birth weight in worldwide. Low birth weight contributes more than 80 percent of all the neonatal mortality. In Ethiopia, studies have shown that there is a high prevalence of low birth weight among newborns. Thus, this study was aimed to determine the magnitude and associated factors with low birth weight among newborns delivered at term in Kambata-Tembaro zone, Southern Ethiopia, 2018.

**Methods:**

Institution based cross-sectional study design was used. The sample size was proportionally allocated to each hospital. The total of 341 study participants was enrolled using systematic random sampling techniques. Data were collected by interview-administered questionnaire and entered using Epi-Info version-7 and exported to SPSS version 20 for analysis. Multivariate logistic regression analysis was carried out to identify associated factors with the low birth weight.

**Results:**

The prevalence of low birth weight was 18% and significantly associated with the mothers’ non-employment [aOR=5.4;95%CI:1.7-17.4], residing in the rural [AOR=5.4; 95%CI:2.1-14.7], unintended pregnancy [aOR=2.0;95%CI:1.2-3.8], not attending antenatal care [aOR=2.3;95%CI: 1.3-2.7], mothers with greater than three births [aOR=1.5;95% CI:1.8-2.6], birth interval less than or equal to two years [aOR=1.9;95%CI:1.6-3.6] and intimate partner violence during pregnancy [aOR=2.1:95% CI: 1.1-3.9].

**Conclusion:**

The study finding shown that the prevalence of low birth weight among newborn was high (18%) in the study. Preventing of low birth weight is an important intervention to reduce neonatal death. Therefore, maximizing women economic status, providing quality family planning services, enabling pregnant women to use antenatal care and preventing intimate partner violence during pregnancy via launching women empowering strategies in the community level is highly recommend.

## Introduction

Low birth weight is defined as a weight of newborns at birth less than 2500 gram by World Health Organization. More than 20 million infants are born with low birth weight in worldwide [1, 2]. Low birth weight is an indicator of a public health problem and also, an important predictor of newborn health and survival [3]. Globally, each year an estimated 2.9million babies die in their first months of life. More than 80% of neonatal mortality occurs among low birth weighted newborns in southern Asia and Sub-Saharan Africa [3, 4]. Birth weight is the most important criteria for determining the neonatal and infants morbidity and mortality. Babies with low birth weight are more vulnerable to risks as compared with normal weighted newborns. It continues to be a significant social problem globally and has both short and long term consequences [5]. From low-middle income countries; systemic reviews revealed that those newborns with low birth weight were increased risk of neonatal morbidity and mortality [6].

Low birth weight is not only a predictor of prenatal morbidity and mortality, but studies have found that it also increases the risk for non-communicable diseases such as diabetes mellitus and cardiovascular diseases in later life [7, 8]. Studies have shown that newborn with low birth weight demonstrates growth retardation and persistent short stature in their life. In most cases, this is linked with abnormal growth hormone secretion and it is associated with poorer school performance [9, 10]. According to Ethiopian demographic health survey 2011, 29.1% newborns were reported as low birth weight and it associated with maternal educational status, lack antenatal care during pregnancy, maternal age at delivery and medical illness during pregnancy [11]. In northwest Ethiopia, studies shown that neonates with low birth weight were two times more likely to die when compared with normal size babies [12, 13]. A study conducted on causes of neonatal mortality in southwest Ethiopia shown that low birth weight babies were two times more likely to die when compared to newborns with weight ≥ 2500g [14]. Study findings revealed that low birth weight was resulted from poor maternal health service utilization preconception and during pregnancy. It is important to ensure that every pregnant woman and newborn have access to good quality care and life-saving interventions [15].

To prevent low birth weight World Health Organization recommended evidence-based maternal health care interventions like women empowerment, women education attainment, appropriate perinatal care, adequate nutrition for adolescent girls, prevention of malaria during pregnancy, birth spacing, antenatal care interventions for all women, iron and folic acid supplements for women during pregnancy daily, interventionist care in severe pre-eclampsia before term and improve linkage and referral for facility births [16, 17]. As literatures review revealed that low birth weight at birth was significantly associated with women illiteracy, woman’s lack of income, women age at birth, early marriage, birth space, maternal illness, maternal reproductive characteristics, women body mass index, personal behaviors, intimate partner violence during pregnancy [18-23]. In Ethiopia, the magnitude of low birth weight among newborns is high and yet, has been contributing a lot to neonatal morbidity and mortality. Even if, much efforts had invested in child health program to tackle the popularity of low birth weight, associated factors and its contribution for neonatal death; still needs further concern and commitments. Moreover, there is still a lack local community-based data on the level of low birth weight and its associated factors that might be vital input for health program designers and all concerned organizations. Addressing factors affecting the birth weight of babies will contribute to the achievement of reducing neonatal mortality in a particular community and nationally. Therefore, the study was aimed to determine the magnitude and factors associated with low birth weight among newborns in Kambata-Tembaro Zone, Sothern Ethiopia, 2018.

## Methods

**Study design and period:** institution-based cross- sectional study was conducted from May 1-30, 2018.

**Source population:** all newborns in the governmental hospitals in Kembata-Tembaro Zone, southern Ethiopia.

**Study population:** all selected newborns in the governmental hospitals in Kembata-Tembaro Zone, southern Ethiopia.

**Inclusion criteria:** all singleton newborns delivered at term, without congenital anomalies were included and newborns from mothers who had diagnosed medical illness during pregnancy were excluded.

**Sample size determination:** the sample size was determined by a single population formula considering the prevalence of low birth weight in Kersa, southern Ethiopia (p=28%) [24], 95% confidence level, 5% margin of error, and considering 10% non-response rate. The total sample size was 341.

**Sampling technique:** the sample size was proportionally allocated to each hospital in the study area and systematic sampling was used to enroll study participants.

**Data collection and quality control:** structured interviewer-administered questionnaire was used to collect data. A tool was developed in English by reviewing different kinds of literature and translated into local language. Questionnaires contained maternal socio-demographic and reproductive characteristics and health care service utilization. Data was collected from postnatal mothers in the health facility at the time of discharge. To ensure quality data, training was given to data collectors and questionnaires were examined for completeness and consistency during data collection. Additionally, data was thoroughly cleaned and carefully entered for the commencement of analysis.

**Data processing and analysis:** data was entered using Epi-Info version-7 and transported to SPSS version 20 software for analysis. Descriptive statistics was done. Multivariable logistic regression analysis was used to identify associated factors with low birth weight newborns at birth and variables with a p-value < 0.05 were declared as significantly associated factors with low birth weight.

**Ethical consideration:** a research ethical approval was obtained from research ethical review committee of the College of Health Science and Medicine, Wachamo University, Ethiopia. A committed was dedicated to evaluating ethical considerations. Written informed consent was obtained from respondents throughout data collection and confidentiality was maintained.

**Ethics approval and consent to participate:** ethical approval was gotten from Wachamo University, College of Health Ethical committee which was dedicated to evaluating ethical consideration of researchers and informed written consent was obtained from study participants during data collections.

## Results

**Socio-demographic characteristics of respondents:** from a total of 341, 334 participants have responded the questionnaires (response rate was 97.9%). The mean age of the respondents’ was found to be 28.68 (SD+5) years. One hundred forty-four (43.1%) of the respondents were couldn’t read and write (Table 1).

**Table 1:** Socio-demographic characteristics of respondents in Kambata-Tembaro Zone Southern Ethiopia, 2018

	Variable	Description	Frequency	Percentage (%)
1	Age in years (Mean age=28.68 (SD+5) years)	≤18	5	1.5
		19-34	266	79.6
		≥ 35	63	18.9
2	Marital status	Married	323	96.7
		Single	5	1.5
		Widowed/divorced	6	1.8
3	Women education status	Can’t read and write	144	43.1
		Primary /secondary school	163	48.8
		Tertiary and above	27	8.1
4	Women occupation	House wife	219	65.6
		Employed	115	34.3
5	Family size (number)	≤5	203	60.8
		>5	131	39.2
6	Residence	Urban	88	26.3
		Rural	246	73.7

**Reproductive characteristics of respondents:** regarding the intention of pregnancy of respondents, 92(27.5%) of pregnancies were unplanned. Concerning antenatal care follows up respondents; 257(76.9%) were attended antenatal care visit; but only 145(43.4%) commenced the first antenatal booking within the first 16-weeks of pregnancy. Eighty-eighty (26.3%) of pregnancies had the history of the birth interval of last pregnancy less than two years (Table 2).

**Table 2:** Reproductive characteristics of respondents in Kambata-Tembaro Zone Southern Ethiopia, 2018

S.N	Variable	Description	Frequency	Percentage (%)
1	Used modern contraceptive	Yes	242	72.5
		No	92	27.5
2	Does the pregnancy was planned (Intention)?	Yes	234	70.1
		No	100	29.9
3	ANC visit	Yes	257	76.9
		No	77	23.1
4	Time to book first ANC visit	≤ 16weeks	145	43.4
		> 16weeks	112	33.5
5	Number of ANC visit	≤3 visit	132	51.4
		> 4 visit	125	48.6
6	Number of birth (parity)	≤ 3 birth	231	69.2
		> 3 birth	103	30.8
7	Birth interval between last birth to previous one	≤ 2 years	88	26.3
		> 2 years	246	73.7
8	History of PROM	Yes	55	16.5
		No	279	83.5
9	IPV during violence	Yes	65	19.5
		No	269	80.5

**Magnitude of low birth weight among newborns delivered at term:** the prevalence of low birth weight among newborns delivered at term was 18% for newborns > 2500g and 82% for newborns ≥ 2500g in the Kambata-Tembaro Zone Southern Ethiopia in 2018.

**Factors associated with the low birth weight of newborns:** multivariate logistic regression analysis revealed that low birth weight of newborn was significantly associated with women occupation, residence, family size, pregnancy intention; antenatal follow up, the number of birth; birth interval and intimate partner violence during pregnancy. Mothers who had no employment were five times more likely to have low birth weight babies as compared to employed mothers [aOR=5.4; 95% CI: 1.7-17.4]. Mothers who had unintended pregnancy were two times more likely to have low birth weight as compared to planned pregnancy [AOR=2; 95% CI: 1.2-3.8]. Mothers who had a history of intimate partner violence during pregnancy were two times more likely to have low birth weight babies as compared to that of mother didn’t have violence during pregnancy [aOR=2.1; 95% CI: 1.1-3.9] (Table 3).

**Table 3:** Factors associated with the low birth weight among newborns delivered at term in Kambata-Tembaro Zone, Southern Ethiopia 2018

		Weight of Newborn				
		<2500g	≥ 2500g			
Variables		Frequency (%)	Frequency (%)	COR 95%CI	*aOR* 95%CI	*P-value*
Women occupation	House wife	41(18.7)	178(81.3)	1.9(1.5-2.6)	5.4(1.7-17.4)	0.004
	Employed	20(17.4)	95(82.6)	Ref	Ref	
Residence	Urban	16(12.5)	112(87.5)	Ref	Ref	
	Rural	45(21.8)	161(78.2)	2(1.05-3.6)	5.4(2.1-14.7)	0.001
Family size	≤ 5	33(16.3)	170(83.7)	Ref	Ref	
	>5	28(21.4)	103(78.6)	1.7(1.4-2.2)	3(1.3-7.4)	0.016
Pregnancy was planned	Yes	36(15.4)	198(84.6)	*Ref*	Ref	
	No	25(25.0)	75(75.0)	1.8(1.03-3.2)	2(1.2-3.8)	0.031
Does ANC visit	Yes	53(20.6)	204(79.4)	*Ref*	Ref	
	No	8(10.4)	69(89.6)	2.2(1.01-4.9)	2.3(1.3-2.7)	0.006
Number of birth (Parity)	≤ 3 births	44 (19.0)	187 (81.0)	*Ref*	Ref	
	> 3 births	17 (16.5)	86 (83.5)	1.2(0.6-2.2)	1.5(1.8-2.6)	0.004
Birth interval (in Years)	≤2	25 (28.4)	63 (71.6)	0.43(0.2-0.7)	1.9(1.6-3.6)	0.031
	> 2	36 (14.6)	210 (85.4)	Ref	Ref	
IPV during pregnancy	Yes	19 (29.2)	46 (70.8)	2.2(1.2-4.1)	2.1(1.1-3.9)	0.025

## Discussion

In this study, the prevalence of low birth weight was found to be 18%. This study finding is comparable with study finding Northwest Ethiopia the prevalence of low birth weight 17.8% [25] and in Southwestern Ethiopia 17.88% [26]. But, in this study finding was lower as compared study done in the Kersa, Ethiopia that the prevalence of Low birth weight was (28.3%) and Ethiopia Demographic health survey 2011 (29.1%) [11, 26].

In contrast, the study finding was higher as compared to other studies result; study done in Northern and southwestern Ethiopia the prevalence of low birth weight was found to be 14.6% and 11.02% respectively [22, 27]. Study done on risk factors for low birth weight in Nigeria showed that the prevalence of 7.3% and the prevalence of low birth weight was 12.3% in study in Kenya [28]. The research result discrepancy might be due to the difference in study time, socio-economic difference in the study population, sample size, and handling of potentially confounding variables.

In this study, mother occupation was significantly associated with low birth weight of newborns. Those mothers who didn’t employed were five times more likely to have low birth weighted babies as compared to mothers who are employed [aOR=5.4; 95% CI: 1.7-17.4]. This might lead housewives to be economically and psychologically dependent on their husbands and affect their health care seeking behavior timely. This woman’s financial dependency is the major hiders of health care seeking behavior in the community.

In this study, mothers residence was found to be significantly associated with low birth weight, meaning mothers residing in the rural area were five times more likely to have low birth weighted babies as compared to mothers from Urban area [aOR=5.4; 95% CI:2.1-14.7]. This finding is consistent with other studies findings [aOR=2.1; 95% CI: 1.04-4.33] and [aOR=0.53; 95% CI: 0.32-0.9] [29] and [aOR=1.43; 95% CI; 1.01-2.01] [30]. This is because the residence might directly and indirectly affect mother health by hindering not to have health care service during pregnancy and may end up adverse birth outcome (LBW).

In this study, mothers who have had unintended pregnancy were two times more likely to have low birth weighted babies as compared to mother with planned pregnancy [aOR=2.0;95%CI:1.2-3.8]. This study finding is consistent with the study finding in the Axum, North Ethiopia with [aOR=4.04; 95% CI: 1.17-13.9] [31]. This might be unintended pregnancy might distress health care seeking behaviors of mothers during pregnancy and exposed for related adverse birth outcome. If the pregnancy is unintended, mothers may have financial shortage which leads to not have adequate nutrition during pregnancy that is necessary for fetal development in the uterus and that might be end up with the adverse birth outcomes.

In this study, the mothers who didn’t attend antenatal care follow up during pregnancy were two times more likely to have low birth weighed babies as compared to mothers who have ANC follow up [aOR=2.3; 95%CI: 1.3-2.7]. This study finding was supported by study done in the Ethiopia, low birth weight was significantly associated with non-attending antenatal care follow up [aOR=1.6; 95% CI: 1.12-2.28] [24]; [aOR=.29; 95% CI: 0.12-0.73] [31], and study done on maternal factors on low birth weight in teaching hospital in Iraq and developing countries [27, 32, 33]. Antenatal care follow up is vitally important to prevent and treat complication during pregnancy that leads low birth weighed newborns and the best strategy to tackle all adverse birth outcomes including low birth weight.

In this study, the number of birth was significantly associated with the low birth weight of babies delivered at term. Mothers who had number of birth greater than three were nearly two times more likely to have low birth weighted babies at term delivery [aOR=1.5; 95%CI: 1.8-2.6]. This finding is in line with finding of study in Mekelle Hospital, Ethiopia and Republic of Indonesia [19, 21]. But, a study in southwestern Ethiopia, the number of birth was not significantly associated with the low birth weight babies at term delivery [27]. As number of birth increases, there is a high chance of having a large family that might affect family socio-economic status.

In this study, mother who gave birth with birth interval less or equal to two year were two times more likely to have low birth weighed babies as compared to those who have three years and above [aOR=1.9; 95%CI: 1.6-3.6]. This is because, if the birth interval is short; it might increase the life risk for mothers related to pregnancy and delivery complications. It might directly or indirectly affects mothers’ health, economic and social status during pregnancy.

In this study, mother who had suffered any act intimate partner violence during pregnancy were two times more likely to have low birth weighed babies as compared to mothers who didn’t have any violence [aOR=2.1: 95% CI: 1.1-3.9]. This study finding is supported by result of study done on intimate partner violence during pregnancy and low birth weight, Southeast Ethiopia [aOR=3.0; 95% CI: 1.57-7.18] [20, 34].

Intimate partner violence might be physical, sexual and psychological which has negative impact on birth weight of newborn. Violence during pregnancy potentially hinders mothers’ health care seeking behaviors, health service utilization and decision making on their all aspects of life. This obviously might lead to have low birth weighted babies.

## Conclusion

The prevalence of low birth weight among newborn was 18% in the study. Prevention of low birth weight is an important intervention to reduce neonatal death. Therefore, to prevent low birth weight among newborns; encouraging women by means of accessing income generating enterprise to maximize their economic status in the community, providing quality and client choose based family planning services, enabling all mothers to use antenatal care during pregnancy by continues and consistent awareness creation and preventing intimate partners violence during pregnancy via empowering women in the community is greatly recommended.

### Limitation of study

In this study, contextual and individual life styles factors that affects weight of newborns didn’t addressed. We recommend other researchers to do follow up study to come up with finds shown that what effects each covariates have by controlling all potential confounders.

### What is known about this topic

- Maternal socio-demographic characteristics like education and age already known factors that associated with low birth weight in most of the studies;
- Maternal medical illness during pregnancy, preterm, multiple pregnancy were already well known associated factors with the low birth weight at birth in most of the studies.

### What this study adds

- In this study, intimate partner violence during pregnancy was significantly associated with the low birth weight among newborns; this was not much revealed by other studies;
- The finding of this study supplemented that appropriate family planning service utilization prevent unintended pregnancy and helps to space birth interval. As result of this study showed unintended pregnancy and short birth interval was significantly associated with low birth weight of newborn. Thus, quality family planning service has great contribution to prevent low birth weight among newborns.

## Competing interests

The authors declare no competing interests.
